# Nuclear Imaging of Glucose Metabolism: Beyond ^18^F-FDG

**DOI:** 10.1155/2019/7954854

**Published:** 2019-03-26

**Authors:** Han Feng, Xiaobo Wang, Jian Chen, Jing Cui, Tang Gao, Yongju Gao, Wenbin Zeng

**Affiliations:** ^1^Department of Pharmacy, Henan Provincial People's Hospital, and People's Hospital of Zhengzhou University, Zhengzhou 450003, China; ^2^Department of Nuclear Medicine, Henan Provincial People's Hospital, and People's Hospital of Zhengzhou University, Zhengzhou 450003, China; ^3^Xiangya School of Pharmaceutical Sciences, and Molecular Imaging Research Centre, Central South University, Changsha 410013, China; ^4^Institute of Nanostructured Functional Materials, Henan Provincial Key Laboratory of Nano-Composite and Application, Huanghe Science and Technology College, Zhengzhou 450006, China

## Abstract

Glucose homeostasis plays a key role in numerous fundamental aspects of life, and its dysregulation is associated with many important diseases such as cancer. The atypical glucose metabolic phenomenon, known as the Warburg effect, has been recognized as a hallmark of cancer and serves as a promising target for tumor specific imaging. At present, 2-deoxy-2-[^18^F]fluoro-glucose (^18^F-FDG)-based positron emission tomography/computed tomography (PET/CT) represented the state-of-the-art radionuclide imaging technique for this purpose. The powerful impact of ^18^F-FDG has prompted intensive research efforts into other glucose-based radiopharmaceuticals for positron emission tomography (PET) and single-photon emission computed tomography (SPECT) imaging. Currently, glucose and its analogues have been labeled with various radionuclides such as ^99m^Tc, ^111^In, ^18^F, ^68^Ga, and ^64^Cu and have been successfully investigated for tumor metabolic imaging in many preclinical studies. Moreover, ^99m^Tc-ECDG has advanced into its early clinical trials and brings a new era of tumor imaging beyond ^18^F-FDG. In this review, preclinical and early clinical development of glucose-based radiopharmaceuticals for tumor metabolic imaging will be summarized.

## 1. Introduction

**Figure 1:**
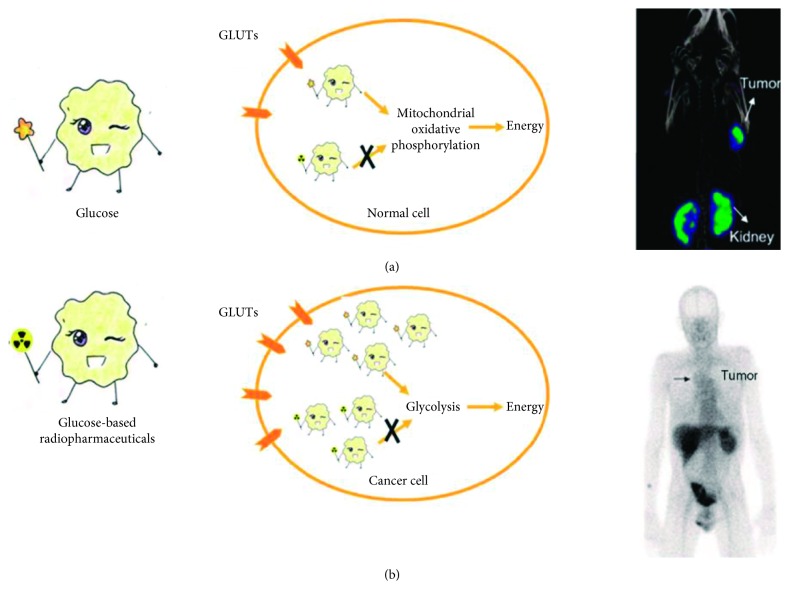
Principles for nuclear imaging of glucose metabolism.

Glucose, a common monosaccharide in nature, is the primary source of energy for most living organisms. Glucose homeostasis plays a key role in numerous fundamental aspects of life, and its dysregulation is associated with many important human diseases such as cancer [1–3]. Cancer is a class of diseases characterized by their uncontrollable proliferation, invasion, and metastasis. In the course of cancer progression, there is a shift of glucose metabolism from mitochondrial oxidative phosphorylation to a glucose-dependent glycolytic pathway, even in the availability of oxygen [4, 5]. To maintain the demand of energy for rapid proliferation, cancer cells increase glucose uptake as well as their glycolytic rate, which can be up to 200 times greater than that of normal cells. This atypical metabolic phenomenon is known as the Warburg effect, which has been recognized as a hallmark of cancer and serves as a promising target for diagnosis and therapy of cancer (Figure 1) [6–8].

At present, 2-deoxy-2-[^18^F]fluoro-glucose (^18^F-FDG)-based positron emission tomography/computed tomography (PET/CT) represents the state-of-the-art radionuclide imaging technique for this purpose. The ^18^F-FDG was synthesized by Pacák and his colleagues in 1969 [9], and it was investigated as a PET tracer in the 1970s and early 1980s [10, 11]. Since then, it has been broadly used in the clinic. Currently, ^18^F-FDG is the most popular glucose-based radiopharmaceutical and is honored as the “molecule of the century” in the field of molecular imaging. The ^18^F-FDG combined with PET/CT has shown great value in the diagnosis, staging, monitoring therapeutic response, and assessment of prognosis [12, 13]. The powerful impact of ^18^F-FDG in the clinic has prompted intensive research efforts into other glucose-based radiopharmaceuticals for positron emission tomography (PET) and single-photon emission computed tomography (SPECT) imaging. In the last decades, glucose and its analogues labeled with various radionuclides such as ^99m^Tc, ^111^In, ^18^F, ^68^Ga, and ^64^Cu have been successfully investigated for tumor metabolic imaging in many preclinical studies [14–17]. Moreover, ^99m^Tc-ECDG has advanced into its early clinical trials and brings a new era of tumor imaging beyond ^18^F-FDG. In this review, preclinical and early clinical development of glucose-based radiopharmaceuticals for tumor metabolic imaging will be summarized.

## 2. Preclinical Development of Glucose-Based Radiopharmaceuticals

### 2.1. SPECT Imaging with ^99m^Tc-Labeled Glucose Analogues

**Figure 2:**
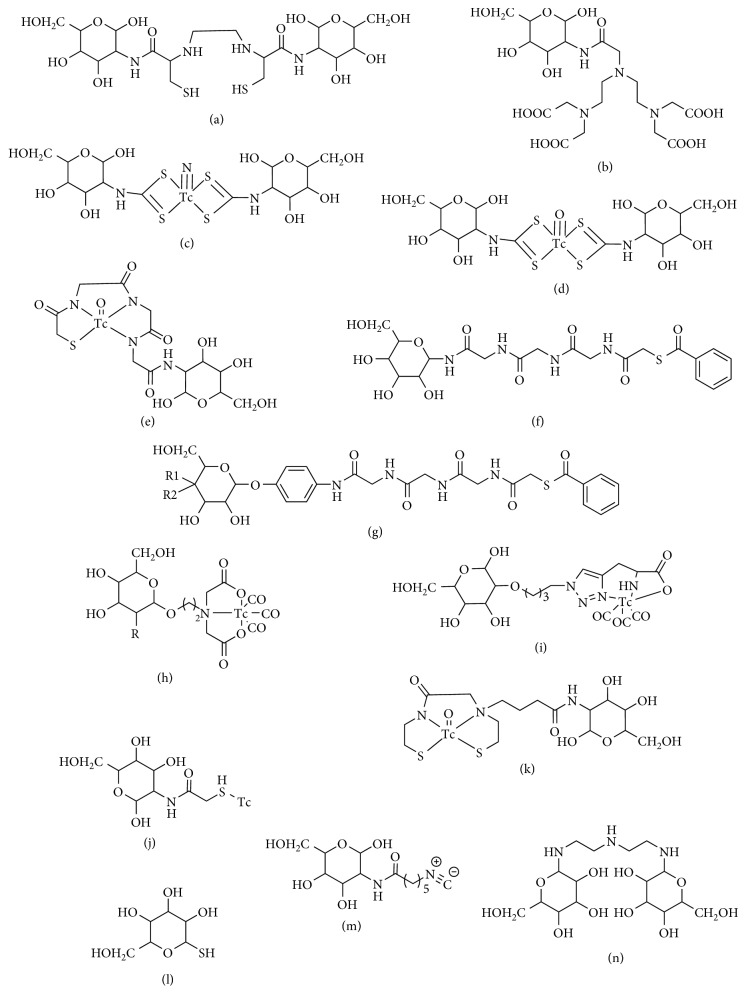
Chemical structures of ^99m^Tc-labeled glucose analogues. (a) ECDG. (b) DTPA-DG. (c) ^99m^TcN-DGDTC. (d) ^99m^TcO-DGDTC. (e) ^99m^Tc-MAG_3_-DG. (f) MAG_3_-G. (g) MAG_3_-GI (R_1_, R_2_, R_3_=H, OH, OH); MAG_3_-Ga (R_1_, R_2_, R_3_=OH, H, OH); MAG_3_-NG (R_1_, R_2_, R_3_=H, OH, NHAc). (h) ^99m^Tc(CO)_3_-IDA-Glucose (R=OH), ^99m^Tc(CO)_3_-IDA-2-Deoxyglucose (R=H). (i) ^99m^Tc(CO)_3_-glucose-histidine. (j) ^99m^Tc-S-DG. (k) ^99m^Tc-MAMA-BA-DG. (l) 1-TG. (m) CN5DG. (n) DGTA.

The availability of commercial generators and kit chemistry to prepare ^99m^Tc-labeled radiopharmaceuticals has had a great impact on nuclear medicine. Considerable efforts have been seen on the development of ^99m^Tc-labeled glucose analogues for tumor imaging. In this section, the summary of these ^99m^Tc-labeled glucose analogues is provided, as shown in Figure 2.

#### 2.1.1. ^99m^Tc-EC-DG

Because of the widely used ^99m^Tc-ethylenedicysteine conjugates, ^99m^Tc-ethylenedicysteine-deoxyglucose (EC-DG) was designed, synthesized, and investigated for tumor imaging by Yang and his colleagues in 2003 [18]. The positive result was achieved in hexokinase assay, which suggested that EC-DG is phosphorylated by hexokinase. The uptake of ^99m^Tc-EC-DG is comparable to that of ^18^F-FDG and was decreased in the presence of D-glucose in lung cancer cells. This finding confirmed the uptake of ^99m^Tc-EC-DG is mediated by glucose transporters. Subsequently, the biodistribution of ^99m^Tc-EC-DG in lung tumor-bearing mice was studied. The results showed the tumor uptake of ^99m^Tc-EC-DG is 2-3 times lower than that of ^18^F-FDG (0.41 ± 0.16 vs 1.60 ± 0.18%ID/g). In view of tumor-to-brain tissue and tumor-to-muscle tissue ratios, ^99m^Tc-EC-DG was superior to ^18^F-FDG. The feasibility of ^99m^Tc-EC-DG for SPECT imaging of tumor was evaluated. The smallest tumor with 3 mm in diameter could be better imaged by ^99m^Tc-EC-DG. The optimized tumor to the corresponding nontumorous region ratio obtained by SPECT was determined to be 1.82 ± 0.07 for the small tumors and 2.88 ± 0.10 for the medium-sized tumors. In addition, the uptake of ^99m^Tc-EC-DG was decreased in rats pretreated with FDG, while it was increased in the group pretreated with insulin, which is consistent with *in vitro* results. These preliminary results demonstrated the potential of ^99m^Tc-EC-DG for tumor metabolic imaging. Currently, ^99m^Tc-EC-DG has advanced into Phase II/III clinical trials (NCT00865319 and NCT01394679), and the corresponding results would be discussed in the following section in detail.

#### 2.1.2. ^99m^Tc-DTPA-DG

In 2006, diethylenetriaminepentaacetic acid-deoxyglucose (DTPA-DG) was synthesized and then labeled with ^99m^Tc using a kit formula with a radiochemical purity of 99.2% [19]. The significant uptake of ^99m^Tc-DTPA-DG was observed at 4 h *in vitro*, which was comparable with that of ^18^F-FDG. The biodistribution of ^99m^Tc-DTPA-DG in breast tumor-bearing rats showed a marked tumor uptake of ^99m^Tc-DTPA-DG, which is 1-2 times higher than that of ^18^F-FDG. Rapid blood clearance of ^99m^Tc-DTPA-DG was visualized with the renal excretion. Compared with ^18^F-FDG, ^99m^Tc-DTPA-DG has higher tumor-to-muscle and tumor-to-brain ratios but a lower tumor-to-blood ratio (4.30 ± 0.89, 19.88 ± 3.45, and 3.24 ± 0.65, respectively). Compared with ^99m^Tc-DTPA, the tumor could be better imaged by ^99m^Tc-EC-DG SPECT imaging with a tumor to nontumor ratio of 2.46 ± 1.02 and 3.54 ± 1.36 at 0.5 and 3 h, respectively. The feasibility of ^99m^Tc-DTPA-DG for tumor imaging has been demonstrated, and ^99m^Tc-DTPA-DG enables visualization of the tumors up to 4 h after injection. In addition, ^99m^Tc-DTPA-DG has been involved in evaluating early chemotherapy response and differentiating the tumor from inflamed tissues [20–23]. Considering these positive results, ^99m^Tc-DTPA-DG may be a potential radiopharmaceutical for tumor imaging. However, the mechanism underlying cellular uptake of ^99m^Tc-DTPA-DG is not clearly understood. Further studies in various animal models and humans are needed.

#### 2.1.3. ^99m^TcN-DGDTC and ^99m^TcO-DGDTC

In 2009, Zhang and his coworkers reported the synthesis of deoxyglucose dithiocarbamate (DGDTC) and radiolabeled it with [^99m^TcN]^2+^ intermediate to prepare ^99m^TcN-DGDTC with a high radiochemical purity (>90%) [24]. ^99m^TcN-DGDTC was demonstrated to be hydrophilic and neutral. The biodistribution study showed the high accumulation of ^99m^TcN-DGDTC in tumors with good retention (1.16 ± 0.57%ID/g at 4 h). Because of the faster blood and muscle clearance, the tumor/blood and tumor/muscle ratios increased with time and reached 2.32 and 1.68 at 4 h after injection. Further studies of the biological characteristics of this radiopharmaceutical may lead to identify a promising candidate for tumor imaging. Similarly, the same group described the radiolabeling of DGDTC by ligand-exchange reaction with ^99m^Tc-glucoheptonate containing the [^99m^TcO]^3+^ core [25]. ^99m^TcO-DGDTC was prepared under neutral condition and at 100°C for 15 min to achieve high radiochemical purity (>90%). ^99m^TcO-DGDTC was hydrophilic and positively charged. The cell uptake of ^99m^TcO-DGDTC increased over time and reached the highest at 4 h. Moreover, the uptake was decreased by the presence of D-glucose, which indicated ^99m^TcO-DGDTC and D-glucose share a similar mechanism of uptake. A significant tumor uptake was observed in biodistribution studies with a long time of retention (2.73 ± 0.72, 2.85 ± 0.63, and 3.53 ± 0.85%ID/g at 0.5 h, 2 h, and 4 h after injection, respectively). Moreover, the tumor-to-blood and tumor-to-muscle ratios were increased over time. As compared with ^99m^TcN-DGDTC, ^99m^TcO-DGDTC had a higher tumor uptake and tumor to muscle ratio but a lower tumor to blood ratio. In contrast, the tumor uptake and tumor-to blood-ratio of ^99m^TcO-DGDTC was lower than that of ^18^F-FDG, but its tumor to muscle ratio is higher. Additionally, the tumor could be clearly detected by SPECT imaging in tumor-bearing mice. These good biological features endow ^99m^TcO-DGDTC as a potential tumor imaging agent.

#### 2.1.4. ^99m^Tc-MAG_3_-Glucose Analogues

As a well-known bifunctional chelator, MAG_3_ has been involved in the development of ^99m^Tc-labeled glucose analogues. In 2006, MAG_3_-DG was designed, synthesized, and radiolabeled via ligand-exchange reaction with ^99m^Tc-glucoheptonate to produce ^99m^Tc-MAG_3_-DG [26]. The biodistribution of ^99m^Tc-MAG_3_-DG was performed in breast tumor-bearing mice. A moderate tumor uptake was observed and estimated to be 0.82 ± 0.06%ID/g. The tumor-to-muscle ratio and tumor-to-blood ratio was determined to be 4.35 ± 1.41 and 0.94 ± 0.13, respectively. In addition, ^99m^Tc-S-DG and ^99m^Tc-MAMA-BA-DG were also synthesized and evaluated in this work. There were significant similarities in the biodistribution of these three radiopharmaceuticals. The only difference is hepatobiliary excretion for ^99m^Tc-MAMA-BA-DG. Among them, ^99m^Tc-MAG_3_-DG showed the most favorable characteristics and could be further studied as potential tumor imaging agents. In 2009, de Barros et al. reported the synthesis of glucose analogue MAG_3_-G and radiolabeled it with ^99m^Tc-tartarate via ligand-exchange reaction [27]. The radiochemical purity was higher than 90%. The biodistribution of ^99m^Tc-MAG_3_-G in Ehrlich tumor-bearing mice showed that the highest tumor uptake (1.64 ± 0.19%ID/g) is obtained at 0.5 h after injection. However, the tumor-to-muscle ratio and tumor-to-blood ratio increased with time and reached 5.03 ± 0.98 and 2.42 ± 0.50 at 8 h after injection. In addition, ^99m^Tc-MAG_3_-G was excreted rapidly through the liver and kidneys. The feasibility of ^99m^Tc-MAG_3_-G for tumor imaging needs to be further evaluated. Similarly, the same group synthesized three compounds MAG_3_-Gl, MAG_3_-Ga, and MAG_3_-NG and successfully radiolabeled them with ^99m^Tc in high radiochemical purities [28]. These three complexes were rapidly excreted through kidneys and had a similar biodistribution in normal mice. Subsequently, the biodistribution of ^99m^Tc-MAG_3_-Gl in tumor mice was carried out [29]. The tumor uptake was high (2.25%ID/g) at 5 min after injection and decreased over time. However, the target-to-nontarget ratio was always greater than 2.0. SPECT imaging showed a marked uptake of ^99m^Tc-MAG_3_-Gl in tumor with a target-to-nontarget ratio of about 2.0, which was in agreement with biodistribution studies. These preliminary results suggested ^99m^Tc-MAG_3_-Gl would possess the potential for tumor imaging.

#### 2.1.5. ^99m^Tc-1-TG and ^99m^Tc-DGTA

As another analogue of *β*-D-glucose, 1-thio-*β*-D-glucose (1-TG) was labeled with ^99m^Tc in high labeling efficiency (>97%) [30]. The *in vitro* assay showed the uptake of ^99m^Tc-1-TG highly depends on 1-TG concentration and was not significantly changed with different glucose concentrations. This finding indicated the tumor uptake mechanism for ^99m^Tc-1-TG was different from that for ^18^F-FDG. Further studies were carried out for early detection of melanoma tumor [31]. The tumor uptake of ^99m^Tc-1-TG was clearly visualized by scintigraphic imaging, showing potential as a new type of radiopharmaceutical for melanoma imaging. In addition, ^99m^Tc-1-TG has been successfully used for inflammation imaging [32].

Lee et al. reported the preparation of diglucosediethylenetriamine (DGTA) from diethylenetriamine and natural D-glucose using a single step chemical synthesis and radiolabeled it with ^99m^Tc in a high radiochemical yield of >95% [33]. The *in vitro* cell uptake of ^99m^Tc-DGTA was 1.5–8 times higher than that of ^18^F-FDG. However, the uptake of ^99m^Tc-DGTA was not highly dependent on glucose concentration, which indicated that its uptake mechanism differs from that of ^18^F-FDG. Although promising, further investigations in animal models are necessary.

#### 2.1.6. ^99m^Tc(CO)_3_-Glucose Analogues

The ^99m^Tc-tricarbonyl ligand (^99m^Tc(CO)_3_) is an interesting tool for ^99m^Tc-labeling techniques [34, 35]. Several types of ^99m^Tc(CO)_3_-labeled glucose analogues have been reported and will be summarized in this section.

In 2010, Ferreira and his colleagues synthesized three carbohydrate-appended 2,2′-dipicolylamine compounds including 2-bis(2-pyridinylmethyl)amino)ethyl-deoxy-1-thio-*β*-D-glucopyranoside (L^**1**^), 2-bis(2-pyridinylmethyl)amino)ethyl-*β*-D-glucopyranoside (L^**2**^), and 2-bis(2-pyridinyl-methyl)amino)carboxamide-*N*-(2-amino-2-deoxy-D-gluco-pyranose) (L^**3**^) and radiolabeled them with [^99m^Tc(CO)_3_]^+^ ligand in high yield (>98%) [36]. Hexokinase assay showed these [^99m^Tc(L^**1–3**^) (CO)_3_]^+^ complexes were not metabolized by hexokinase. The biodistribution of [^99m^Tc(L^**1−3**^) (CO)_3_]^+^ complexes in tumor mice demonstrated an initial high uptake and a rapid elimination from tumor with time. From this point of view, these [^99m^Tc(L^**1−3**^) (CO)_3_]^+^ complexes are not suitable for tumor imaging. Subsequently, the hydrophilic iminodiacetic acid (IDA) was covalently coupled with C-modified glucose and 2-deoxyglucose, which was then labeled with [^99m^Tc(CO)_3_]^+^ ligand to form the corresponding ^99m^Tc(CO)_3_-IDA-glucose and ^99m^Tc(CO)_3_-IDA-2-deoxyglucose in high yield [37, 38]. The tumor cell uptake of ^99m^Tc(CO)_3_-IDA-glucose and ^99m^Tc(CO)_3_-IDA-2-deoxyglucose was observed with the highest internalization of 18% and 52% of the total activity, respectively. However, only the uptake of ^99m^Tc(CO)_3_-IDA-Glucose was decreased by the presence of D-glucose, which indicated the uptake of ^99m^Tc(CO)_3_-IDA-Glucose is mediated by glucose transporters. These two radiopharmaceuticals were rapidly excreted through the kidneys and accumulated in urine and the bladder with time, which is consistent with their hydrophilicity. Moreover, the tumor uptake of ^99m^Tc(CO)_3_-IDA-glucose and ^99m^Tc(CO)_3_-IDA-2-deoxyglucose were observed and decided to be 0.31 ± 0.23 and 0.40 ± 0.28%ID/g at 1 h after injection, respectively. The corresponding tumor-to-muscle ratios and tumor-to-blood ratios were estimated to be 2.5 ± 0.3 and 0.21 ± 0.05 for ^99m^Tc(CO)_3_-IDA-glucose and 2.0 ± 0.7 and 0.24 ± 0.02 for ^99m^Tc(CO)_3_-IDA-2-deoxyglucose, respectively. These favorable features endow ^99m^Tc(CO)_3_-IDA-glucose and ^99m^Tc(CO)_3_-IDA-2-deoxyglucose as promising imaging agents, which justifies further investigations in animal models and humans.

Additionally, Fernández et al. reported the derivatization of glucose at C-2 using the so-called “click chemistry” to form a histidine-like, 1,4-disubstituted triazole molecule for radiolabeling with [^99m^Tc(CO)_3_]^+^ ligand [39]. A relatively low protein binding of ^99m^Tc(CO)_3_-glucose-histidine was obtained, correlating with its high *in vitro* stability and hydrophilicity. Biodistribution was characterized by low blood and liver uptake. Because of its hydrophilicity, renal excretion was observed as expected. The tumor uptake of ^99m^Tc(CO)_3_-glucose-histidine was moderate and retained for a long time. The tumor-to-muscle ratio was high and was determined to be 2.75 ± 0.06 at 2 h after injection. By comparison, ^99m^Tc(CO)_3_-glucose-histidine and ^18^F-FDG have a similar biodistribution in C57BL/6 mice bearing induced Lewis murine lung carcinoma. However, the tumor uptake and tumor-to-muscle ratio of ^99m^Tc(CO)_3_-glucose-histidine are much lower than those of ^18^F-FDG. Modifications of the structure are needed to improve biological properties.

#### 2.1.7. ^99m^Tc-CN5DG

**Figure 3:**
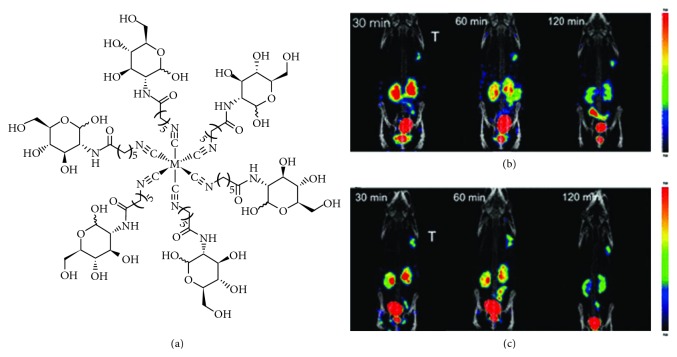
A novel ^99m^Tc-labeled glucose derivative for SPECT imaging. (a) Chemical structure of ^99m^Tc-CN5DG. Whole-body SPECT/CT images of ^99m^Tc-CN5DG (55 MBq) in nude mice bearing A549 xenografts with a tumor size of 3 mm (b) and 5 mm (c) in diameter at 30 min, 60 min, and 120 min after injection. Adapted from Reference [40] with permission.

Recently, a D-glucosamine derivative with an isonitrile group (CN5DG) was synthesized and labeled with ^99m^Tc to prepare ^99m^Tc-CN5DG (Figure 3) [40]. ^99m^Tc-CN5DG was readily obtained with high radiochemical purity (>95%) and specific activity (11.17–335.22 GBq/mmol). This hydrophilic radiopharmaceutical exhibited great *in vitro* stability and metabolic stability in urine. The tumor cell uptake of ^99m^Tc-CN5DG was significantly blocked in the presence of D-glucose and increased by insulin, which demonstrated that ^99m^Tc-CN5DG is transported via glucose transporters. Biodistribution studies in mice bearing A549 xenografts showed that ^99m^Tc-CN5DG had a rapid, high tumor uptake and cleared quickly from normal organs, resulting in a satisfactory tumor-to-background ratio. The tumor uptake of ^99m^Tc-CN5DG is comparable to that of ^18^F-FDG. However, the tumor-to-blood, tumor-to-muscle, and tumor-to-lung ratios of ^99m^Tc-CN5DG are much higher than those of ^18^F-FDG (19.83 ± 4.39 vs 8.40 ± 3.89, 14.37 ± 6.96 vs 0.32 ± 0.08, and 5.22 ± 0.58 vs 1.19 ± 0.29) at 1 h after injection. Furthermore, ^99m^Tc-CN5DG coupled clearly visualizes the tumor sites for a long time. The smallest tumor that can be detected by ^99m^Tc-CN5DG SPECT imaging was about 3 mm. These excellent biological characteristics confirmed that ^99m^Tc-CN5DG may be a potential “working horse” and be another breakthrough in glucose-based radiopharmaceuticals for tumor imaging.

### 2.2. SPECT Imaging with ^111^In-Labeled Glucose Analogues

The *γ*-emitting radioisotope indium-111 (^111^In) (*t*_1/2_ = 2.83 d, 171 KeV (90%), 245 KeV (94%)) is of great practical interest for clinical SPECT [41–43]. Because of its large size, the macrocyclic chelators such as diethylenetriaminepentaacetic acid (DTPA), 1,4,7,10-tetraazacyclodode cane-1,4,7,10-tetraacetic acid (DOTA), and 1,4,7-triazacyclononane-1,4,7-triacetic acid (NOTA) have usually been used to form stable metal complexes for further conjugation of biomolecules [44]. In 2012, Yang and his coworkers reported the development of radiopharmaceutical ^111^In-DOTA-DG from precursor compound DOTA-DG and ^111^In with the labeling efficiency of >95% and radiochemical purity of >96% (Figure 3) [45]. Nude mice bearing MDA-MB-468 mammary tumors were employed to evaluate the pharmacokinetics and targeting ability of ^111^In-DOTA-DG. The prominent accumulation of radioactivity in the liver, kidneys, and urinary bladder was observed, indicating that this radiopharmaceutical is mainly through the renal excretory pathway. As seen from the SPECT images, the tumors were visualized at 120 min after injection. The results suggest that ^111^In-DOTA-DG may be a promising glucose-based radiopharmaceutical for SPECT imaging. Further detailed evaluation is required to elucidate its metabolic mechanism.

### 2.3. SPECT Imaging with Radioiodine-Labeled Glucose Analogues

**Figure 4:**
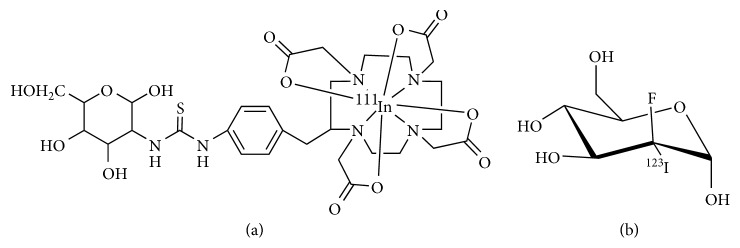
Chemical structures of ^111^In-DOTA-DG (a) and ^123^I-FIM (b).

Because of the excellent physical properties, easy accessibility, and low manufacturing cost, radioiodination of glucose analogues for SPECT imaging is of great clinical interest. So far, several radioiodine-labeled glucose analogues have been proposed [46, 47]. Iodine-123 (^123^I) is the halogen isotope of choice due to its excellent physical properties that make it ideal for imaging. Of them, 2-deoxy-2-[^123^I] iodo-glucose (IDG) is the most logical form of iodinated glucose analogues. However, this small molecule is not stable under physiological conditions, making it unsuitable for imaging applications [46]. Subsequently, 2-deoxy-2-fluoro-2-iodo-D-mannose (FIM) and 2-deoxy-2-fluoro-2-iodo-D-glucose (2-FIG) were synthesized and shown to be stable for several days in saline, which demonstrated that the presence of fluorine on position 2 enables the stability of iodine atom in glucose analogues [48]. Therefore, the FIM was labeled with ^123^I to produce 2-fluoro-2-[^123^I]iodo-D-mannose (^123^I-FIM), which was stable *in vitro* for 24 hours (Figure 4). Unfortunately, *in vivo* studies showed ^123^I-FIM has a rapid blood clearance and high stomach and thyroid uptake, indicating its rapid deiodination after injection [49]. In addition, iodination of glucose isomers in positions 3, 4, and 6 were investigated, and none of them exhibited the similar biological features of 2-deoxy-D-glucose [50]. The iodinated glucose analogues have not been found to be metabolic markers for *in vivo* studies.

### 2.4. PET Imaging with ^18^F-Labeled Glucose Analogues

**Figure 5:**
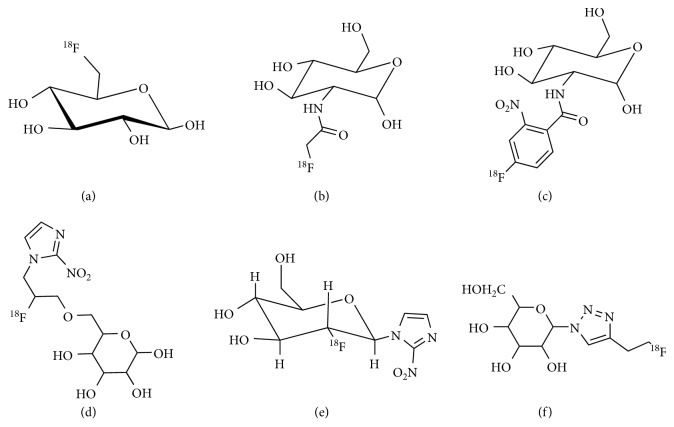
Chemical structures of ^18^F-labeled glucose analogues. (a) ^18^F-6FDG. (b) ^18^F-FAG. (c) ^18^F-FNBG. (d) ^18^F-GAZ. (e) ^18^F-FDG-2-NIm. (f) 4-[(2-[^18^F]fluoroethyl)-1-(D-glucopyranosyl)]-1H-1,2,3-triazole.

The ^18^F-FDG is the only Food and Drug Administration- (FDA-) approved glucose-based radiopharmaceutical and has been used worldwide. The success of ^18^F-FDG leads to the development of other ^18^F-labeled glucose analogues for tumor imaging (Figure 5).

#### 2.4.1. ^18^F-6FDG

With the goal of developing the radiopharmaceuticals similar to ^18^F-FDG, 6-deoxy-6-[^18^F]fluoro-D-glucose (^18^F-6FDG) was synthesized and prepared, starting with D-glucose, in 60–70 min with a decay-corrected yield of 71 ± 12% and radiochemical purity of ≥96% [51]. The preliminary *in vitro* and *in vivo* studies demonstrated ^18^F-6FDG may be a more representative candidate for the glucose transporter than ^18^F-FDG. Interestingly, because of the substitution of fluorine at C-6 position, ^18^F-6FDG is just transported through glucose transporters and cannot be phosphorylated for subsequent metabolism [52]. Meanwhile, 1-[^18^F]fluorodeoxyfructose, 1-[^18^F]-fluoroalkyldeoxyglucose, and other glucose analogues have been evaluated as novel candidates for PET imaging [53].

#### 2.4.2. ^18^F-Labeled Glucosamine

*N*-[^18^F]Fluoroacetyl-D-glucosamine (^18^F-FAG) was the first D-glucosamine analogue to be radiolabeled with ^18^F by Fujiwara and his colleagues in 1990 [54]. C3H/HeMsNRS mice with spontaneous hepatomas were used for PET imaging. The high uptakes of ^18^F-FAG were observed in the tumor, liver, and kidney at 60 min after injection, whose mean value was estimated to be 5.16%ID/g, 3.71%ID/g, and 3.27%ID/g, respectively. Among all tissues, the tumor has the highest radioactivity with a long retention (5.51%ID/g at 5 min after injection and 5.16%ID/g at 60 min after injection). Furthermore, the tumor was clearly visualized by PET imaging in the rabbit VX-2 tumor model. Therefore, ^18^F-FAG is a promising PET radiotracer for tumor imaging. Another glucosamine-based radiopharmaceutical, of note, is *N*-(2-[^18^F]fluoro-4-nitrobenzoyl)glucosamine (^18^F-FNBG) [55]. The biodistribution study in mice models showed ^18^F-FNBG mainly accumulates in the tumor, liver, and kidney. The tumor uptake of ^18^F-FNBG became the highest at 5 min after injection with a value of 1.68 ± 0.05%ID/g and was decreased with time. At 120 min, the tumor still has an uptake of 0.21 ± 0.02%ID/g, which is comparable to that of the liver and kidney at the same time. Besides, the tumor uptake of ^18^F-FNBG was 0.44 ± 0.12%ID/g at 60 min, while it was 4.32 ± 0.79%ID/g for ^18^F-FAG. However, the tumor/blood and tumor/muscle ratios of ^18^F-FNBG were similar with those of ^18^F-FAG. In addition, Carroll et al. reported the synthesis of three novel ^18^F-labeled glucosamine analogues (^18^F-**5**, ^18^F-**8** and ^18^F-**13**) and the evaluation of their tumor uptake *in vivo* by PET imaging [56]. Among them, ^18^F-**13** showed a discernible tumor uptake of 2.80 ± 0.51%ID/g at 60 min after injection, which is 5.12 ± 1.59%ID/g for ^18^F-FDG. In view of these primary results, ^18^F-labeled glucosamine analogues might be promising candidates for tumor PET imaging. More studies are needed to further investigate their imaging property.

#### 2.4.3. ^18^F-FDG-2-NIm and ^18^F-GAZ

In 2002, Patt and his coworkers synthesized a new glucose-coupled 2-nitroimidazole derivative, ^18^F-FDG-2-NIm, from the peracetylated 2-[^18^F]FDG in good radiochemical yields [57]. In comparison of ^18^F-FDG, *in vitro* and *in vivo* studies demonstrated much lower uptake of ^18^F-FDG-2-NIm, which suggested that the accumulation into tumor cells *via* glucose transporters is unlikely to occur. Another similar radiopharmaceutical is an azomycin-glucose conjugate ^18^F-GAZ [58]. PET imaging showed the accumulation of ^18^F-GAZ was observed at 5-6 min after injection (0.66 ± 0.05%ID/g) and decreased in a time-dependent manner. At 60 min after injection, the tumor uptake was measured to be 0.24 ± 0.04%ID/g with a tumor/muscle ratio of 1.87 ± 0.18. However, competitive experiment showed F-GAZ is a weaker competitive inhibitor of ^18^F-FDG compared with D-glucose and unlabeled 2-FDG. ^18^F-GAZ seems not to be uptaken by glucose transporters, and further studies should be carried out.

#### 2.4.4. 4-[(2-[^18^F]Fluoroethyl)-1-(*β*-D-glucopyranosyl)]-1H-1,2,3-triazole

In 2008, Kim et al. developed an ^18^F-labeled glucose analogue, 4-[(2-[^18^F]fluoroethyl)-1-(*β*-D-glucopyranosyl)]-1H-1,2,3-triazole, via click reaction between glucopyranosyl azide and 4-[^18^F]fluoro-1-butyne [59]. In comparison with conventional routes, the click labeling method spent shorter time to obtain the radiopharmaceutical with higher decay-corrected radiochemical yield and specific activity. Unfortunately, this radiopharmaceutical was demonstrated to be incompatible for hexokinase phosphorylation and independent of glucose transporter.

### 2.5. PET Imaging with ^11^C-Labeled Glucose Analogues

Because carbon is a component of biomolecules, labeling with the positron-emitting radioisotope carbon-11 (^11^C) seems to be very attractive. Many attempts have been made to label glucose with radioisotope ^11^C [60, 61]. [2-^11^C]-2-Deoxyglucose was developed as the ^11^C counterpart of ^18^F-FDG [62]. Unfortunately, this radiotracer is not metabolically trapped. Subsequently, numerous radiopharmaceuticals such as 6-[^11^C]-d-glucose and 1-[^11^C]-d-mannitol have been obtained by labeling glucose derivates with ^11^C-Wittig reagent, ^11^C-cyanide, or ^11^C- nitromethane [63–67]. However, the radiolabeling process is the multistep chemical manipulation and time-consuming, which is incompatible with the short half-life of ^11^C (*t*_1/2_ = 20.3 min). Therefore, direct ^11^C labeling strategies are still needed. In 2003, Bormans et al. developed a nonmetabolizable ^11^C-labeled *α*/*β*-methyl-D-glucoside (^11^C-*α*MDG and ^11^C-*β*MDG) that is selectively transported by sodium dependent glucose transporters (SGLTs) [68]. These radiotracers were prepared by straightforward methylation of glucose with ^11^C-methyl triflate in a total synthesis time of 20 min and a yield of 30% (decay corrected). *In vivo* PET imaging showed ^11^C-labeled *α*/*β*-methyl-D-glucoside accumulated in the kidney, which depends on the functionality of SGLTs in the luminal membrane of renal proximal tubules. Consequently, ^11^C-methyl-D-glucoside is a promising PET tracer for the *in vivo* visualization of SGLTs in kidney malfunction. Future studies are needed to elucidate whether ^11^C-methyl-D-glucoside may be used to detect various human tumors with high level of SGLT transporters.

### 2.6. PET Imaging with ^68^Ga-Labeled Glucose Analogues

The growth and worldwide spread of positron emitting radionuclide gallium-68 (^68^Ga) in preclinical and clinical research has proven its potential for PET imaging during last two decades. The advantages of ^68^Ga such as favorable physical and chemical properties, commercially available generators, robust labeling chemistry diversity have been presented in detail in many literatures and strongly motivate researchers to develop new ^68^Ga-based radiopharmaceuticals [69, 70].

**Figure 6:**
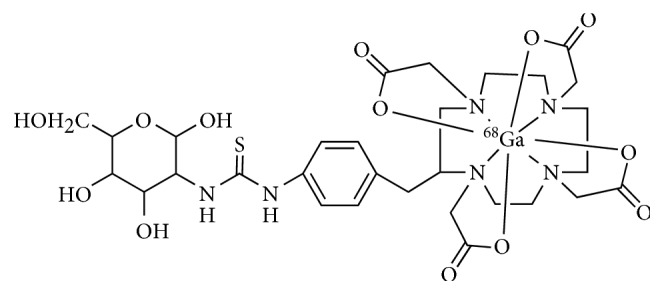
Chemical structures of ^68^Ga-DOTA-DG.

In 2012, Yang et al. radiolabelled 1,4,7,10-tetraazacyclododecane-1,4,7,10-tetraacetic acid (DOTA)-2-deoxy-D-glucosamine (DOTA-DG) with ^68^Ga to produce radiopharmaceutical ^68^Ga-DOTA-DG with the labeling efficiency of ∼85% and radiochemical purity of 98% in ten minutes assisting with microwave (Figure 6) [71]. The percentage uptake of ^68^Ga-DOTA-DG in A431 cells at 60 min is comparable to that of ^18^F-FDG (15.7% and 16.2%, respectively). In a human tumor xenograft model, the tumor uptake of ^68^Ga-DOTA-DG was determined to be 0.39%ID/g, which is much lower than that of ^18^F-FDG (4.26%ID/g) after 60 min injection. However, the tumor-to-heart, tumor-to-brain, and tumor-to-muscle ratios are higher in comparison with ^18^F-FDG. Additionally, the PET images showed renal excretion and accumulation in the bladder. Significant researches are needed to elucidate the potential of ^68^Ga-DOTA-DG as a candidate for clinical tumor imaging.

### 2.7. PET Imaging with ^64^Cu-Labeled Glucose Analogues

Compared with positron emitting radionuclide ^18^F and ^11^C, the long half-life (*t*_1/2_ = 12.7 h), decay profile (*E*_*β*+_ = 653 KeV (18%), E_*β*−_ = 579 KeV (38.4%), EC (44.6%)), and well-established coordination chemistry of ^64^Cu make it more suitable for labeling of biological molecules and PET imaging [72–75].

**Figure 7:**
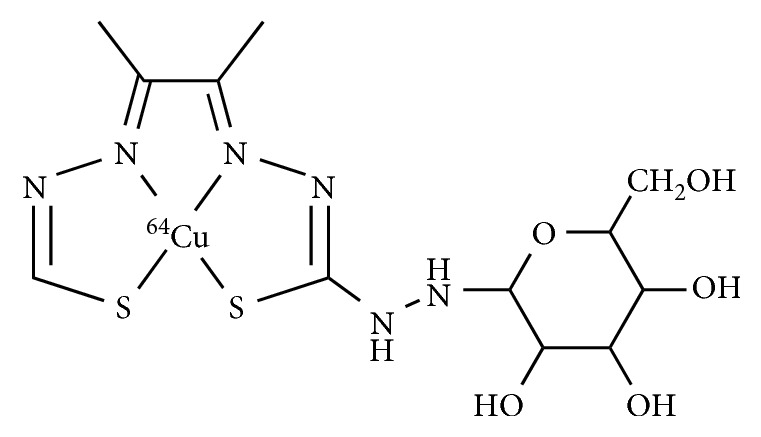
Chemical structures of ^64^Cu-ATSE/A-G.

The precursor Zn-ATSE/A-G was synthesized and radiolabeled by ^64^Cu *via* copper-zinc exchange with a radiochemical yield of 71.7% (Figure 7) [76, 77]. In the PET images, ^64^CuATSE/A-G displayed moderate tumor uptake and a divergent pattern of biodistribution compared with ^18^F-FDG. Renal excretion and accumulation in the bladder were observed. In particular, the distinctive brain and heart uptake of ^18^F-FDG was not obtained in the images of ^64^Cu-ATSE/A-G, which demonstrate that it does not participate in glucose-specific transport and is not a surrogate for glucose metabolism imaging. In addition, the uptake of ^64^Cu-ATSE/A-G in proportion to the O_2_ concentration in the HeLa cells demonstrated its hypoxia selectivity and feasibility for hypoxia imaging.

## 3. Early Clinical Development of Glucose-Based Radiopharmaceuticals

**Figure 8:**
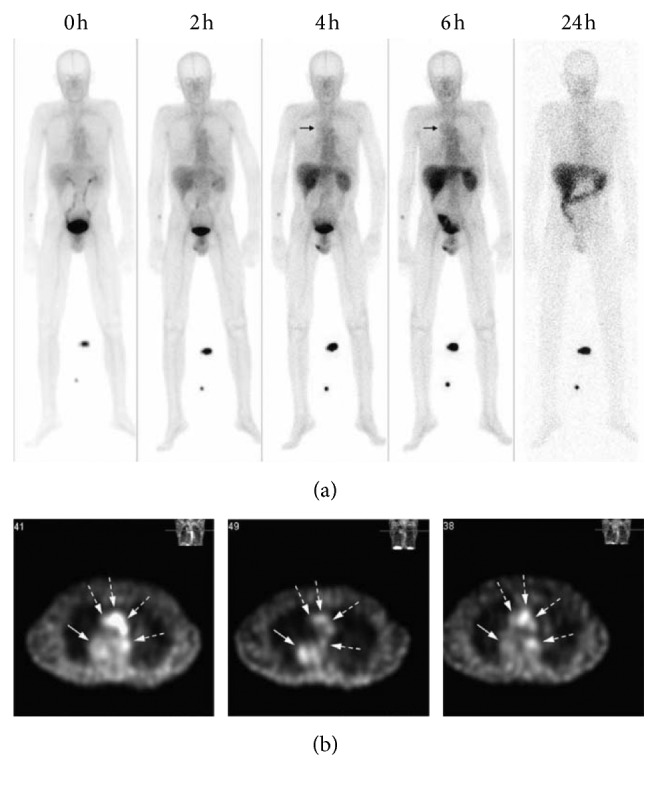
Radiation dosimetry and biodistribution of ^99m^Tc-EC-DG in patients with non-small-cell lung cancer. (a) Whole-body images of ^99m^Tc-EC-DG in a patient with NSCLC in the right upper lung (arrow), obtained after administration of 925 MBq (25 mCi). The images were acquired immediately and 2, 4, 6, and 24 h after injection. (b) CT attenuation-corrected SPECT transverse slices obtained after administration of 925 MBq (25 mCi) of ^99m^Tc-EC-DG in a patient with NSCLC in the medial posterior right upper lung (solid arrow). Uptake is also seen in the blood pool (great vessels; dashed arrows). The three images (left to right) are from the 2, 4, and 6 h SPECT scans, respectively. Adapted from Reference [78] with permission.

Since 1969, when ^18^F-FDG was developed for PET imaging in the clinic, intense attempts have been made in the development of other glucose-based radiopharmaceuticals for SPECT and PET imaging, which create a pipeline of exciting tracers for tumor imaging. Among them, ^99m^Tc-EC-DG is the only glucose-based radiopharmaceutical, which has advanced in Phase II/III clinical trials (NCT00865319, NCT01394679). To assess the biodistribution, radiation dosimetry, and diagnostic efficacy, ^99m^Tc-EC-DG SPECT imaging and ^18^F-FDG PET imaging were performed in seven patients with non-small-cell lung cancer (NSCLC) (Figure 8) [78]. It was found that the uptake of ^99m^Tc-EC-DG was mainly visualized in the blood pool, kidneys, bladder, and liver over time. Bladder wall was deemed to be the critical organ that receives the highest dose, with an average radiation absorbed dose of 2.47 × 10^−2^ mGy/MBq. The mean effective dose equivalent and effective dose were estimated to be 6.20 × 10^−3^ mSv/MBq and 5.90 × 10^−3^ mSv/MBq for administration of 1,110 MBq activity, which is less than that of ^18^F-FDG (3.00 × 10^−2^ mSv/MBq). Whole-body images showed that the primary tumor was clearly visualized in six of the seven patients at 4 h after injection with confirmed NSCLC and concordant accumulation of ^18^F-FDG. However, tumor-to-background ratios obtained with ^99m^Tc-EC-DG is lower than that of ^18^F-FDG. The patient with negative uptake of ^99m^Tc-EC-DG and positive uptake of ^18^F-FDG was pathologically documented to have a granuloma. In addition, the administration of ^99m^Tc-EC-DG was well tolerated in this cohort of patients. All these results are encouraging and endow ^99m^Tc-EC-DG as a diagnostic agent for nuclear medicine imaging. Larger scale clinical studies are now warranted to assess the utility of ^99m^Tc-EC-DG for tumor imaging.

## 4. Conclusions

Diagnosis, staging, monitoring therapeutic response, and assessment of prognosis in the management of patients with cancer pose major challenges to today's medical imaging. The development of ^18^F-FDG PET imaging represents a major milestone in the field of molecular imaging to overcome these limitations. Along a similar line, glucose and its analogues have been chemically modified by a carefully researched bifunctional chelator with their biochemical properties retained, labeled with various radionuclides, and explored for tumor imaging. Among these glucose-based radiopharmaceuticals for SPECT imaging, ^99m^Tc-EC-DG, ^99m^Tc-MAG_3_-DG, and ^99m^Tc-CN5DG represent a few named diagnostic tracers in this field. Novel glucose-based PET radiopharmaceuticals, such as ^18^F-6FDG, ^18^F-GAZ, and ^68^Ga-DOTA-DG, substantiate their advantages over others, showing great potential for clinical translation. These radiopharmaceuticals bring a new era of tumor imaging beyond ^18^F-FDG. Despite a wealth of preclinical data, only ^99m^Tc-EC-DG has advanced in Phase II/III clinical trials, and the cases reported so far are few in number. The barriers for developing promising glucose-based radiopharmaceuticals and preventing the translation of them in the clinic are many and not clear. The first challenge lies in the choice of radiochemistry. The introduction of a prosthetic or bulky metal-bearing moiety may have a large impact on the overall biochemical properties of a glucose analogue. Therefore, the structure-activity relationship of glucose-based radiopharmaceuticals should be carefully optimized. The simple and fast radiolabeling processes with a high radiochemical purity and specific activity are essential for the development of a promising radiopharmaceutical. In addition, the “theranostics” is new direction of nuclear medicine. Researchers should put efforts to explore the labeling of glucose analogues with ^177^Lu, ^90^Y, ^188^Re, etc. and to investigate them as potential theranostic agents. On the other hand, the design of preclinical studies and clinical trials for clinical translation of glucose-based radiopharmaceuticals are also very important. Therefore, the concerted efforts from pharmacologists, radiologists, oncologists, and clinicians are required to validate these well-designed radiopharmaceuticals as clinical diagnostic agents. Furthermore, it is our belief that the increasing radiolabeled glucose analogues will enter clinical trials, progress to authorized approval, and ultimately become widely used imaging agents in the clinic.
